# Clinical benefit of immune checkpoint inhibitors approved by US Food and Drug Administration

**DOI:** 10.1186/s12885-020-07313-2

**Published:** 2020-08-31

**Authors:** Fei Liang, Sheng Zhang, Qin Wang, Wenfeng Li

**Affiliations:** 1 Medical Oncology, Shanghai Cancer Center, Fudan University, 270 Dongan Road, Shanghai, 200032 China; 2 Department of Biostatistics, Zhongshan Hospital, Fudan University, Shanghai, China; 3 Shanghai University of Engineering Science, Shanghai, China; 4 Department of Medical oncology, the affiliated hospital of Qingdao University, Qingdao, China

**Keywords:** Randomized trials, Clinical benefits, Immune checkpoint inhibitors, Cancer, Food and drug administration agency

## Abstract

**Background:**

We describe the clinical benefit of immune checkpoint inhibitors using the European Society for Medical Oncology Magnitude of Clinical Benefit Scale (ESMO-MCBS) and ASCO VF.

**Methods:**

We identify all approved indications of immune checkpoint inhibitors based on RCTs between January 1, 2011 and September 30, 2018 by FDA. Information including medians and HR of OS (PFS or DFS) and 95% CI, grade 3 or 4 toxicities in each arm, QOL data, survival probability at fixed time were extracted.

**Results:**

Immune checkpoint inhibitors were approved for 18 indications based on RCTs. All the indications meet the ESMO-MCBS 1.1 threshold for meaningful benefit. By the updated ASCO-VF, the median Net Health Benefit (NHB) of these agents was 55.3 (range 17.4–77.1). Two third of the indication gained the bonus points for durable survival benefits by updated ASCO VF. When updated results were incorporated in the assessment, clinical benefit of most approved immune checkpoint inhibitors increased with a median improvement of NHB of 10 (range 2–20).

**Conclusions:**

Approved immune checkpoint inhibitors provided clinical meaningful benefit by ESMO-MCBS 1.1, and most of these agents reach the threshold for bonus points for durable survival in the updated ASCO VF.

## Background

Knowledge of the potential benefits and risks associated with the use of anticancer therapies is fundamental for making treatment-related recommendations and decisions. Two important oncology societies have recently taken a step forward to quantize the clinical benefit. The American Society of Clinical Oncology (ASCO) Value Framework (ASCO-VF) [1], which was updated in 2016 [2], and the European Society for Medical Oncology developed its Magnitude of Clinical Benefit Scale (ESMO-MCBS) for drugs indicated in the treatment of solid cancer [3], which also updated in 2017 [4]. They have been used to grade US Food and Drug Administration (FDA)-approved new drugs for treating advanced solid cancers [5–7]. In the study by Vivot and colleagues, they found that Many recently FDA-approved new cancer drugs did not have high clinical benefit as measured by ASCO-VF and ESMO-MCBS.

The growing wave of progress using cancer immunotherapy, which has extended and improved the lives of patients, many of whom had few other effective treatment options has yielded high expectations from all stakeholders. However, there are also concerns about the value of check point inhibitors. Many immune checkpoint inhibitors were approved based on single-arm studies, only recently more RCTs were finished and reported.

Patient-reported outcomes (PROs), such as symptoms, quality of life (QOL), and patient-perceived health status supplement clinical data and are now more important during decision-making in oncology because they provide a holistic understanding of patient experience and treatment effectiveness [8, 9]. Both ESMO-MCBS and ASCO VF incorporated QOL into the determination of the value of a treatment. ASCO VF awarded bonus points for treatment with a statistically significant improvement in cancer-related symptoms. However, PROs usually were not reported in the primary report or approval documents, but subsequently reported as separate articles.

In this study, we aimed to describe the clinical benefit of checkpoint inhibitors that were recently approved by the FDA based on RCTs using ESMO-MCBS and ASCO VF, and whether these agents reach defined thresholds of long-term benefit in the two value frameworks. We also compare the values based on primary reports with those assessed based on updated reports including long-term survival reports and/or QOL reports.

## Methods

### Data sources

We identify all approved indications of immune checkpoint inhibitors (Ipilimumab, Nivolumab, pembrolizumab, Atezolizumab, Avelumab, and Durvalumab, Cemiplimab) between January 1, 2011 and September 30, 2018 by searching FDA website [10]. Only indications approved based on RCTs were included and those approved based on single arm trials were excluded. Indications that were granted accelerated approval based on single arm trials but subsequently obtained regular approval with positive confirmatory RCTs were included. We included drugs used both in the metastatic setting and adjuvant setting of treatment of solid tumors.

### Data extraction

Information including medians and HR of OS (PFS or DFS) and 95% CI, grade 3 or 4 toxicities in each arm, QOL data, survival probability at fixed or specified time were extracted from the reports of pivotal clinical trials supporting the FDA approval and FDA documents (drug labels and review summary retrieved from Drugs@fda website [10]). Survival probability at fixed or specified time was extracted directly from Kaplan-Meier curves using digital software (DigitizeIt). Baseline characteristics such as drug name, indication, trial name, sample size, primary outcome, tumor type, year of approval were also collected. When statistically significant results were reported for more than experimental arms, then each arm was evaluated separately and assigned a separate grade.

### ESMO-MCBS and the ASCO-VF

ASCO-VF and ESMO-MCBS both quantify treatment benefit in a survival endpoint. ESMO-MCBS grade was assigned based on the lower limit of the 95% confidence interval of the hazard ratio (HR), and in conjunction with the minimum absolute gain differences in median survival or by the increase in survival at a fixed time, and further adjusted on QOL, toxicity and long term plateau of survival curve. ESMO-MCBS grades, in the non-curative setting, range from 1 to 5, with grades 4 and 5 representing meaningful clinical benefit, in the curative setting, range from A to C, with A and B representing meaningful clinical benefit. ASCO-VF score was assigned primarily on the point estimate of the HR with adjustment on toxicity and bonus points including tail of the curve, palliation, QOL and treatment-free interval. ASCO-VF score is continuous with a higher score representing a better score, and no cut-off value was provided to define clinical benefit. Both value frameworks incorporated amendments to introduce tail-of-the curve credits for progression-free survival and overall survival. For ESMO-MCBS, credit is given for a 10% or greater absolute gain at prognostically weighted specified time points in the true tail of the curve. Grading based on “long term” survival points differs depending on a PFS or OS endpoint (i.e., for PFS, this is an upgrade, while, for OS, this is an additional grading using the curative framework, e.g., 4/A). None of the trials actually meet this OS upgrade given the length of time required for the data to mature. ASCO-VF awarded 20 points of tail-of-the-curve bonus points if, at twice the median survival time (or DFS) in the control arm, there was an improvement of at least 50% in survival provided the survival in the control group was at least 20% and award 16 points (0.8 × 20) if the improvement is in PFS. ASCO-VF further awarded treatment with a statistically significant improvement in cancer-related symptoms (10 points) or improvement in treatment-free interval (10 points).

Two review authors (F.L. and S.Z.) independently scored each indication using ESMO-MCBS and the ASCO-VF with discrepancies resolved by a third investigator. We used the k coefficient to determine degree of agreement between reviewers. For trials with two or more immunotherapy arms, we scored each arm separately, but only the arm with higher score was used to represent the value of the specific indication in all analysis.

### Updated value score

Value of approved drugs may change as long-term follow-up data or QOL data (which is usually not available or reported when initially approved) become available. Particularly, drugs that failed to qualify the tail of the curve bonus due to limited follow-up time can show long term plateauing of survival with longer follow-up time.

We searched latest drug label or PubMed to identify if updated reports of survival, toxicity or quality of life data and assigned updated score for these indications. When multiple reports of updated reports of survival were published, the most up-to-date one was used.

## Results

Eighteen indications for 5 immune checkpoint inhibitors were approved by the FDA for metastatic solid tumors based on RCTs from March 2011 to September 2018 (Table 1). Two approvals were for adjuvant therapy and 16 for non-curative therapy. The approvals were for melanoma (7 indications), NSCLC (7 indications), head and neck cancer (1indication), urothelial carcinoma (1indication) and renal cell carcinoma (1indication). Median sample size of pivotal RCTs was 694 (range 272–1034) (Table 1).

**Table 1:** Characteristics of immune-checkpoint inhibitors approved by US FDA

Approved drug	Indications	Pivotal Trial	Primary endpoint	Sample size	Year of approval
Pembrolizumab plus chemotherapy	First-line therapy of metastatic non-squamous NSCLC	KEYNOTE-189	OS and PFS	616	2018
Nivolumab plus ipilimumab	First-line therapy of intermediate or poor risk advanced renal cell carcinoma	CHECKMATE-214	OS, ORR and PFS	847	2018
Durvalumab	Consolidation therapy for stage III NSCLC who did not have disease progression after two or more cycles of platinum-based chemoradiotherapy	PACIFIC	PFS and OS	713	2018
Atezolizumab	Second line therapy of NSCLC	OAK	OS	850	2016
Pembrolizumab	Second line therapy foradvanced urothelial carcinoma	KEYNOTE-45	PFS and OS	542	2017
Pembrolizumab	First-line NSCLC with tumors express PD-L1 > 50% as determined by an FDA-approved test	KEYNOTE-24	PFS	305	2017
Pembrolizumab	Second line therapy of metastatic NSCLC whose tumors express PD-L1	KEYNOTE-010	PFS and OS	1034	2016
Pembrolizumab	First-line therapy of melanoma	KEYNOTE-006	PFS and OS	834	2015
Nivolumab	Adjuvant therapy of melanoma	CHECKMATE-238	RFS	906	2017
Nivolumab	Second line therapy of squamous-cell carcinoma of the head and neck	CHECKMATE-141	OS	361	2016
Nivolumab	Second line therapy of renal cell carcinoma	CHECKMATE-025	OS	821	2015
Nivolumab	Second line therapy of advanced squamous-cell NSCLC	CHECKMATE-017	OS	272	2015
Nivolumab	Second line therapy of advanced nonsquamous NSCLC	CHECKMATE-057	OS	582	2015
Nivolumab	First line therapy of BRAF wild-type unresectable or metastatic melanoma	CHECKMATE-066	OS	418	2015
Nivolumab with or without ipilimumab	First line therapy of unresectable or metastatic melanoma	CHECKMATE-067	PFS and OS	945	2015
Pembrolizumab	Second line therapy of unresectable or metastatic melanoma	KEYNOTE-002	PFS	540	2015
Ipilimumab	Adjuvant therapy of melanoma	EORTC-18071	RFS	951	2015
Ipilimumab	Second line therapy of unresectable or metastatic melanoma	MDX010–20	OS	676	2011

*FDA* Food and Drug Administration, *NSCLC* Non–small cell lung cancer, *PD-L1* Programmed death-ligand 1, *OS* Overall survival, *PFS* Progression-free survival, *RFS* Recurrence-free survival

### Clinical benefit of immune checkpoint inhibitors

Eighteen pivotal RCTs were included for the value assessment, with 5 trials had two experimental arms. By the ESMO-MCBS 1.1, for the 16 trials in the non-curative setting, 8 trials were grade five (the highest), and 8 trials grade four. For the two trials in the adjuvant setting, both were grade A. Thus, all trials met the ESMO-MCBS meaningful benefit threshold. Three trials met the ESMO-MCBS long term benefit criteria, all with the primary endpoint of PFS. Twelve of trials meet the criteria of improved toxicity (less grade 3–4 toxicities impacting on daily well-being) and only one trial was considered as increased toxic death.

**Fig. 1:**
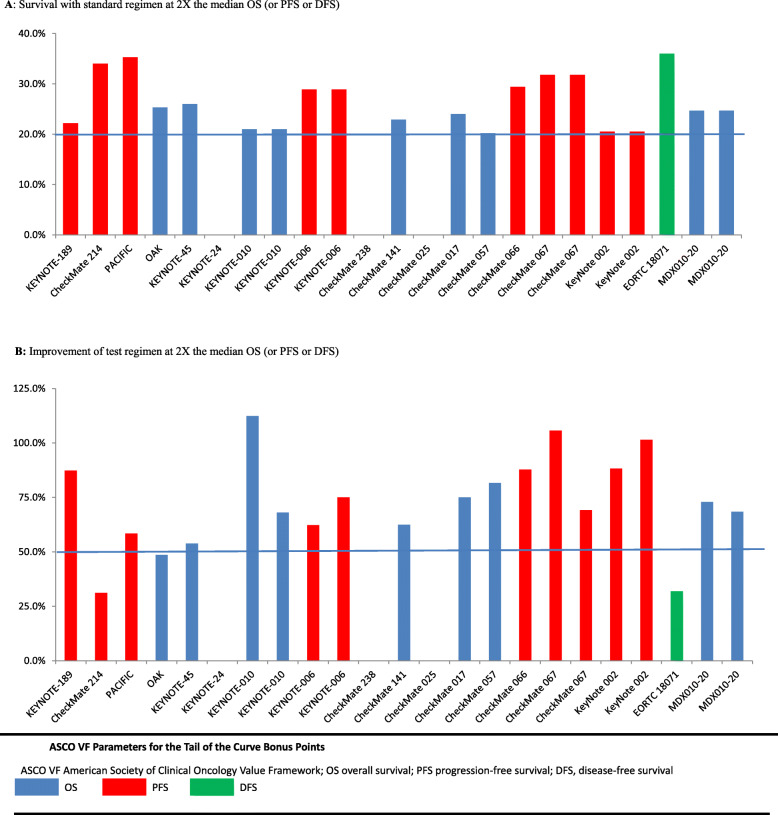
ASCO VF Parameters for the Tail of the Curve Bonus Points

By the ASCO-VF, the median Net Health Benefit (NHB) of drugs was 55.3 (range 17.4–77.1). The median treatment effect score was 34.4 (range 25–58) and the median toxicity score was 3.8 (range − 7.6 to 11.3), with 13 trials have positive toxicity score and 5 trials with negative toxicity score (Table 2). 12(66.7%) trials gained the long tail bonus points in the ASCO framework. Bonus points for a tail on OS curves were granted for 6 trials (33.3%) and for PFS curves for 6 trials (33.3%) (Fig. 1). For the remaining 6 trials not qualified for the tail of the curve bonus, survival proportions with standard regimen at 2X the median OS (or PFS or DFS) were not available for three trials due to limited follow-up time and three trials did not achieved the required 50% improvement in patients alive in the test regimen compared with the standard (Fig. 1). Bonus for palliation symptoms was granted for 1 trial (5.5%); and for improvement in QoL for 3 trials (16.7%). No drugs received bonus points for treatment-free interval (Table 2).

**Table 2:** ESMO-MCBS and ASCO VF scores of Pivotal RCTs of immune-checkpoint inhibitors approved by US FDA

Trial name	Evaluated endpoint	ESMO-MCBS					ASCO VF								
		HR (95% CI)	QOL	Toxicity	Long term benefit	Score	Clinical benefit score		Toxicity score	Tail of the curve	Palliation		QOL	Treatment-free interval	NHB
KEYNOTE-189	OS	0.49 (0.38–0.64)	Not reported	Not improved	Not qualified	4	51		−7.5	16	0		0	0	59.5
CHECKMATE-214	OS	0.63 (99.8%CI, 0.44–0.89	Improved	Improved	Not qualified	5	37		4.9	0	0		10	0	51.9
PACIFIC	PFS	0.52(0.42 to 0.65)	Not reported	Not improved	> 10% improvement in PFS at 1 year	4	38.4		−6.7	16	0		0	0	47.7
OAK	OS	0.73 (0.62–0.87)	Not reported	Improved	Not qualified	5	27		2.7	0	0		0	0	29.7
KEYNOTE-45	OS	0.73 (0.59–0.91)	Not reported	Improved	Not qualified	4	27		1.0	20	0		0	0	48.0
KEYNOTE-24	OS	0.60 (0.41–0.89)	Not reported	Improved	Not qualified	5	40		5.9	0	0		0	0	45.9
KEYNOTE-010-1^a^	OS	0.61 (0.49–0.75)	Not reported	Improved	Not qualified	5	39		5.7	20	0		0	0	64.7
KEYNOTE-010-2^b^	OS	0.71 (0.58–0.88)	Not reported	Improved	Not qualified	3	29		6.6	20	0		0	0	55.6
KEYNOTE-006-1^c^	OS	0.69 (0.52–0.90)	Not reported	Improved	Not qualified	5	31		2.3	16	0		0	0	49.3
KEYNOTE-006-2^d^	OS	0.63 (0.47–0.83)	Not reported	Improved	Not qualified	5	37		3.1	16	0		0	0	56.1
CHECKMATE-238	RFS	0.65 (0.51–0.83)	Not Improved	Improved	Not qualified	A	35		3.8	0	0		0	0	38.8
CHECKMATE-141	OS	0.70 (0.52, 0.92)	Improved	Improved	Not qualified	4	30		7.1	20	10		10	0	77.1
CHECKMATE-025	OS	0.73 (98.5% CI, 0.57–0.93)	Improved	Improved	Not qualified	5	27		6.8	0	0		10	0	43.8
CHECKMATE-017	OS	0.59 (0.44–0.79)	Not reported	Improved	Not qualified	5	41		11.3	20	0		0	0	72.3
CHECKMATE-057	OS	0.73 (0.60–0.89)	Not reported	Improved	Not qualified	5	27		8.3	20	0		0	0	55.3
CHECKMATE-066	OS	0.42 (99.79% CI, 0.25–0.73)	Not Improved	Not Improved	Not qualified	4	58		2.4	16	0		0	0	76.4
CHECKMATE- 067-1^e^	PFS	0.42 (99.5% CI, 0.31 to 0.57)	Not reported	Increased toxic death	> 10% improvement in PFS at 1 year	3	46.4		−5.1	16	0		0	0	57.3
CHECKMATE-067-2^f^	PFS	0.57 (99.5% CI, 0.43 to 0.76)	Not reported	Improved	> 10% improvement in PFS at 1 year	4	34.4		5.5	16	0		0	0	55.9
KEYNOTE-002-1^g^	PFS	0.57 (0.45–0.73)	Not reported	Improved	> 10% improvement in PFS at 1 year	4	34.4		7.3	16	0		0	0	57.7
KEYNOTE-002-2^h^	PFS	0.50 (0.39–0.64)	Not reported	Improved	> 10% improvement in PFS at 1 year	4	40		5.9	16	0		0	0	61.9
EORTC-18071	RFS	0.75 (0.64–0.90)	Not improved	Not improved	Not qualified	A	25		−7.6	0	0		0	0	17.4
MDX010–20-1^i^	OS	0.66 (0.51–0.87)	Not reported	Not improved	Not qualified	4	34		1.0	20	0		0	0	55.0
MDX010–20-2^j^	OS	0.68 (0.55–0.85)	Not reported	No improved	Not qualified	4	32		−0.34	20	0		0	0	51.7
Trial name	Endpoint	Drug	Cancer	ESMO-MCBS					ASCO VF						
				HR (95% CI)	QOL	Toxicity	Long term benefit	Score	Clinical benefit score	Toxicity score	Tail of the curve	Palliation	QOL	Treatment-free interval	NHB
KEYNOTE-189*	OS	Pembrolizumab	NSCLC	0.49 (0.38–0.64)	Not reported	Not improved	Not qualified	4	51	−7.5	16	0	0	0	59.5
CHECKMATE-214*	OS	Nivolumab	RCC	0.63 (99.8%CI, 0.44–0.89	Improved	Improved	Not qualified	5	37	4.9	0	0	10	0	51.9
PACIFIC*	PFS	Durvalumab	NSCLC	0.52(0.42 to 0.65)	Not reported	Not improved	> 10% improvement in PFS at 1 year	4	38.4	−6.7	16	0	0	0	47.7
OAK	OS	Atezolizumab	NSCLC	0.73 (0.62–0.87)	Not reported	Improved	Not qualified	5	27	2.7	0	0	0	0	29.7
KEYNOTE-45	OS	Pembrolizumab	UC	0.73 (0.59–0.91)	Not reported	Improved	Not qualified	4	27	1.0	20	0	0	0	48.0
KEYNOTE-24	OS	Pembrolizumab	NSCLC	0.60 (0.41–0.89)	Not reported	Improved	Not qualified	5	40	5.9	0	0	0	0	45.9
KEYNOTE-010-1^a*^	OS	Pembrolizumab	NSCLC	0.61 (0.49–0.75)	Not reported	Improved	Not qualified	5	39	5.7	20	0	0	0	64.7
KEYNOTE-010-2^b*^	OS	Pembrolizumab	NSCLC	0.71 (0.58–0.88)	Not reported	Improved	Not qualified	3	29	6.6	20	0	0	0	55.6
KEYNOTE-006-1^c^	OS	Pembrolizumab	Melanoma	0.69 (0.52–0.90)	Not reported	Improved	Not qualified	5	31	2.3	16	0	0	0	49.3
KEYNOTE-006-2^d^	OS	Pembrolizumab	Melanoma	0.63 (0.47–0.83)	Not reported	Improved	Not qualified	5	37	3.1	16	0	0	0	56.1
CHECKMATE-238	RFS	Nivolumab	Melanoma	0.65 (0.51–0.83)	Not Improved	Improved	Not qualified	A	35	3.8	0	0	0	0	38.8
CHECKMATE-141	OS	Nivolumab	SCCHN	0.70 (0.52, 0.92)	Improved	Improved	Not qualified	4	30	7.1	20	10	10	0	77.1
CHECKMATE-025*	OS	Nivolumab	RCC	0.73 (98.5% CI, 0.57–0.93)	Improved	Improved	Not qualified	5	27	6.8	0	0	10	0	43.8
CHECKMATE-017	OS	Nivolumab	NSCLC	0.59 (0.44–0.79)	Not reported	Improved	Not qualified	5	41	11.3	20	0	0	0	72.3
CHECKMATE-057*	OS	Nivolumab	NSCLC	0.73 (0.60–0.89)	Not reported	Improved	Not qualified	5	27	8.3	20	0	0	0	55.3
CHECKMATE-066*	OS	Nivolumab	RCC	0.42 (99.79% CI, 0.25–0.73)	Not Improved	Not Improved	Not qualified	4	58	2.4	16	0	0	0	76.4
CHECKMATE- 067-1^e*^	PFS	Nivolumab	Melanoma	0.42 (99.5% CI, 0.31 to 0.57)	Not reported	Increased toxic death	> 10% improvement in PFS at 1 year	3	46.4	−5.1	16	0	0	0	57.3
CHECKMATE-067-2^f*^	PFS	Nivolumab	Melanoma	0.57 (99.5% CI, 0.43 to 0.76)	Not reported	Improved	> 10% improvement in PFS at 1 year	4	34.4	5.5	16	0	0	0	55.9
KEYNOTE-002-1^g^	PFS	Pembrolizumab	Melanoma	0.57 (0.45–0.73)	Not reported	Improved	> 10% improvement in PFS at 1 year	4	34.4	7.3	16	0	0	0	57.7
KEYNOTE-002-2^h^	PFS	Pembrolizumab	Melanoma	0.50 (0.39–0.64)	Not reported	Improved	> 10% improvement in PFS at 1 year	4	40	5.9	16	0	0	0	61.9
EORTC-18071*	RFS	Ipilimumab	Melanoma	0.75 (0.64–0.90)	Not improved	Not improved	Not qualified	A	25	−7.6	0	0	0	0	17.4
MDX010–20-1^i*^	OS	Ipilimumab	Melanoma	0.66 (0.51–0.87)	Not reported	Not improved	Not qualified	4	34	1.0	20	0	0	0	55.0
MDX010–20-2^j*^	OS	Ipilimumab	Melanoma	0.68 (0.55–0.85)	Not reported	No improved	Not qualified	4	32	−0.34	20	0	0	0	51.7

*ESMO-MCBS* European Society for Medical Oncology Magnitude of Clinical Benefit Scale, *ASCO VF* American Society of Clinical Oncology Value Framework, *RCT* Randomized controlled trial, *FDA* Food and Drug Administration, *HR* Hazard ratio, *CI* Confidence interval, *QOL* Quality of life, *NHB* Net health benefit, *OS* Overall survival, *PFS* Progression-free survival, *RFS* Recurrence-free survival, *NSCLC* Non–small-cell lung cancer, *RCC* Renal-cell carcinoma, *UC* Urothelial carcinoma, *SCCHN* Squamous cell carcinoma of the head and neck

*: Reported only adverse events that occurred in at least 10% of the treated patients

^a^ Pembrolizumab 10 mg/kg arm in the KEYNOTE-010 trial

^b^ Pembrolizumab 2 mg/kg arm in the KEYNOTE-010 trial

^c^ Pembrolizumab every 3 weeks arm in the KEYNOTE-006 trial

^d^ Pembrolizumab every 2 weeks arm in the KEYNOTE-006 trial

^e^ Nivolumab plus ipilimumab arm in the CHECKMATE-067 trial

^f^ Nivolumab arm in the CHECKMATE-067 trial

^g^ Pembrolizumab 2 mg/kg arm in the KEYNOTE-002 trial

^h^ Pembrolizumab 10 mg/kg arm in the KEYNOTE-002 trial

^i^ Iplimumab arm in the MDX010–20 trial

^j^ Ipilimumab plus gp100 arm in the MDX010–20 trial

For trials with ESMO-MCBS grade of 4, the median NHB was 49.3 (range 17.4–72.3), while for those with ESMO-MCBS grade of 5 or A, the median NHB was 56.8 (range 47.7–71.1).

### Updated clinical benefit

Fourteen trials reported updated survival results or toxicity data or quality of life data. ESMO-MCBS grades were changed for two trials, both of which increased from 4 to 5 (Table 3). The ESMO-MCBS grade of CHECKMATE-066 [11, 12], which support the approval of nivolumab as first line therapy of BRAF wild-type unresectable or metastatic melanoma increased from 4 to 5 due to improved QOL, which were not available in the primary report [11] and approval documents. Nivolumab as second line therapy of squamous-cell carcinoma of the head and neck obtained ESMO-MCBS grade of 4 based on lower limit of HR of OS < 0.65 and gain of 2.4 months (preliminary score of 3) and improved QOL and less grade 3–4 toxicities reported in the primary report of CHECKMATE-141 trial [13]. This indication now obtained a score of 5 due to increased preliminary score with increase in 2 year survival of > 10% reported in the 2-year long-term survival update report [14]. Two trials (PACIFIC and CHECKMATE-067) no longer met the ESMO-MCBS long term benefit criteria when evaluated with subsequently reported OS results instead of PFS (Table 3). Both trials were first evaluated using PFS due to immature OS results and met the criteria of long term PFS benefit with > 10% improvement. When undated mature OS results were available, they were re-evaluated using OS and did not meet the criteria of long term OS benefit that OS advantage continues to be observed at 7 years.

**Table 3:** Updated ESMO-MCBS and ASCO VF scores of Pivotal RCTs of immune-checkpoint inhibitors with updated results

Trial name	Evaluated endpoint	ESMO-MCBS					ASCO VF						
		HR (95% CI)	QOL	Toxicity	Long term benefit	Score	Clinical benefit score	Toxicity score	Tail of the curve	Palliation	QOL	Treatment free interval	NHB
PACIFIC-Updated^a^	OS	0.68 (99.73% CI, 0.47–0.997)	Not reported	Not improved	Not qualified	4	32	−6.2	16	0	0	0	41.8
OAK-Updated	OS	0.75 (0.64–0.89)	Not improved	Improved	Not qualified	5	25	2.7	0	10	0	0	37.7
KEYNOTE-45-Updated	OS	0.73 (0.59–0.91)	Improved	Improved	Not qualified	4	27	1.0	20	10	10		68.0
KEYNOTE-24-Updated	OS	0.60 (0.41–0.89)	Improved	Improved	Not qualified	5	40	5.9	0	10	10	0	65.9
KEYNOTE-006-1-Updated	OS	0.68 (0.53–0.87)	Improved	Not improved	Not qualified	5	32	2.3	16	0	10	0	60.3
KEYNOTE-006-2-Updated	OS	0.68 (0.53–0.86)	Improved	Not improved	Not qualified	5	32	3.1	16	0	10	0	61.1
CHECKMATE-141-Updated	OS	0.68 (0.54–0.86)	Improved	Improved	Not qualified	5	32	7.1	20	10	10	0	79.1
CHECKMATE-025-Updated^a^	OS	0.73 (98.5% CI, 0.57–0.93)	Improved	Improved	Not qualified	5	27	6.8	0	10	10	0	53.8
CHECKMATE-017-Updated	OS	0.62 (0.48–0.80)	Improved	Improved	Not qualified	5	38	11.3	20	10	10	0	89.3
CHECKMATE-057-Updated^a^	OS	0.73 (0.62–0.88)	Improved	Improved	Not qualified	5	27	8.3	20	10	10	0	75.3
CHECKMATE-066-Updated^a^	OS	0.42 (99.79% CI, 0.25–0.73)	Improved	No Improved	Not qualified	5	58	2.4	16	0	10	0	86.4
CHECKMATE-067-1-Updated^a^	OS	0.54 (0.44–0.67)	Not Improved	Increased	Not qualified	3	46	−9.0	20	0	0	10	67.0
CHECKMATE-067-2-Updated^a^	OS	0.65 (0.53–0.79)	Not Improved	Not improved	Not qualified	4	35	0.3	20	0	0	0	55.3
KEYNOTE-002-1-Updated	PFS	0.57 (0.45–0.73)	Improved	Improved	> 10% improvement in PFS at 1 year but without OS benefit	4	34.4	5.4	16	10	10	0	75.8
KEYNOTE-002-2-Updated	PFS	0.50 (0.39–0.64)	Improved	Improved	> 10% improvement in PFS at 1 year but without OS benefit	4	40	4.4	16	10	10	0	80.4
EORTC-18071-Updated^a^	OS	0.72 (0.58–0.88)	Not improved	Not improved	Not qualified	A	28	−8.5	0	0	0	0	19.5
MDX010–20-1-Updated^a^	OS	0.66 (0.51–0.87)	No improved	No improved	Not qualified	4	34	1.0	20	0	0	0	55.0
MDX010–20-2-Updated^a^	OS	0.68 (0.55–0.85)	No improved	No improved	Not qualified	4	32	−0.3	20	0	0	0	51.7

*ESMO-MCBS* European Society for Medical Oncology Magnitude of Clinical Benefit Scale, *ASCO VF* American Society of Clinical Oncology Value Framework, *RCT* Randomized controlled trial, *FDA* Food and Drug Administration, *HR* Hazard ratio, *CI* Confidence interval, *QOL* Quality of life, *NHB* Net health benefit, *OS* Overall survival, *PFS* Progression-free survival, *RFS* Recurrence-free survival

^a^Reported only adverse events that occurred in at least 10% of the treated patients

**Fig. 2:**
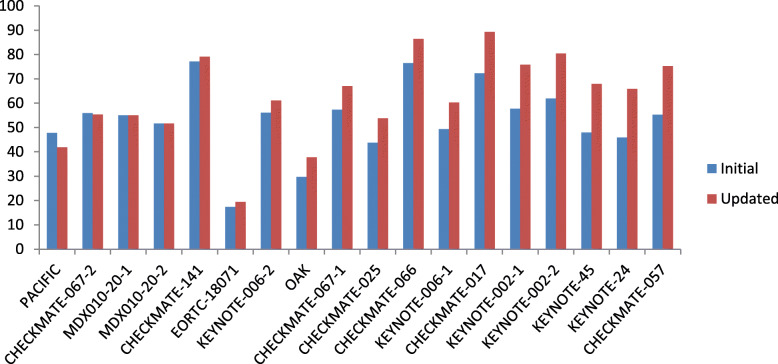
Comparison of ASCO VFs evaluated based on initial reports and updated reports

By the ASCO-VF, the NHB were changed for 13 trials with updated results (Fig. 2, Table 3). One indication, durvalumab as consolidation therapy for stage III NSCLC, obtained a NHB of 47.7 with initial PFS results [15] but obtained an updated NHB of 41.8 based on the OS results [16]. For the rest of 13 trials, the NHB based on the updated reports is improved because of the awarding of bonus points for a statistically significant improvement in the QoL (7 trials) or/and statistically significant improvement in cancer-related symptoms (7 trials) and/or statistically significant improvement in treatment-free interval (1 trial). The median improvement of NHB was 10 (range 2–20). The maximum 20 increase of NHB were seen in three indications: pembrolizumab as second line therapy for advanced urothelial carcinoma (KETNOTE-045 trial) [17, 18], pembrolizumab as first-line NSCLC with tumors express PD-L1 > 50% as determined by an FDA-approved test (KETNOTE-024 trial) [19, 20], and nivolumab as second line therapy of advanced nonsquamous NSCLC (CHECKMATE-057 trial) [21, 22].

## Discussion

A previous study by Vivot A et al. [7], which assessed the clinical benefit of new drugs for treating advanced solid tumors aproved by the US FDA between 2000 and 2015 using ASCO-VF and ESMO-MCBS, reported that 13 (35%) out of 51 approved anticancer drugs showed a meaningful clinical benefit (scale levels 4 and 5) by ESMO-MCBS, and the median drug value was 37 (interquartile range 3.4–67) by ASCO-VF. Another study by Tibau A et al. [5] evaluated the magnitude of clinical benefit of cancer drugs approved by the US FDA from January 2006 to December 2016 using ESMO-MCBS, and found that fewer than half of RCTs supporting FDA approval meet the threshold for clinically meaningful benefit. However, less than 20% of the approved agents were immune checkpoint inhibitors in these studies, with more than 60% of approved agents being target therapy.

In our analysis, all trials met the ESMO-MCBS meaningful benefit threshold and by the ASCO-VF, the median NHB of drugs was 55.3 (range 17.4–77.1). Although caution should be taken in interpreting across study comparisons, due to the fact we used updated ASCO-VF and ESMO-MCBS, the clinical benefit seems greater in immune checkpoint inhibitors than other approved cancer drugs. Only two trials in the adjuvant setting were included in our study. Both trial meet the ESMO-MCBS 1.1 threshold for meaningful benefit. NHB of the two agents were 37.8 and 17.4, which seems lower than those in the metastatic setting. Further studies are need to evaluate whether the clinical benefit of immune checkpoint inhibitors in the adjuvant setting is consistent with those in the metastatic setting with more agents were approved in the adjuvant setting.

Recently, Ben-Aharon et al. [23], tried to determine whether immuno-oncology agents approved by the FDA fulfill the durable survival threshold defined in the updated ASCO-VF. They found only 3 drug indications fulfilled the threshold. However, in our study, 12 of 18 approved indications gained the bonus points for durable survival benefits. Several issues may explain the discrepancies. First,, as pointed by Vivot et al. in their letter [24] to the editor and Schnipper et al. in their commentary [25], Ben-Aharon et al. used raw proportions of patients at risk (ie, number of patients still at risk divided by the number of patients randomized) to estimate the survival proportion instead of using the probability displayed on Kaplan-Meier curves, which may have may have disqualify trials that actually met the ASCO-VF criteria for long term benefit.. Second, only 10 indications approved based on RCTs were eligible for their analysis. Only recently more RCTs of immunotherapy have been finished and reported. And they were never evaluated with ESMO-MCBS. Our study provided important and comprehensive evaluation of approved immune checkpoint inhibitors in RCTs.

Although our study did not aim to or was powered to assess the consistency of updated ASCO-VF and the ESMO-MCBS 1.1 due to limited number of RCTs included, Clinical benefits by updated ASCO-VF and the ESMO-MCBS 1.1 yielded some sorts of consistencies. For trials with ESMO-MCBS grade of 5 or A, the median NHB was numerical higher than those with ESMO-MCBS grade of 4. A recent study [26] that evaluated the concordance between the two frameworks in the noncurative setting showed that agreement between the frameworks was higher than observed in other studies that sought to compare them [27, 28]. This study was done by the authorship group of the two frameworks (vs independent groups). Concordance will likely be greater when those individuals who created the value frameworks are the ones scoring/grading. Another cohort comparing the two frameworks has also drawn similar conclusions [29]. The issue of framework utility in the general oncology community has been raised recently [30].

We found that 12 of 18 indications gained the bonus points for tails of the curve, while only 3 indications met the ESMO-MCBS long term benefit criteria, all with the primary endpoint of PFS. This discrepancy is not surprising given the differences in their criteria. To qualify for the long-term plateau by ESMO-MCBS 1.1 [4], overall survival advantage need to be observed at 5 years if the median overall survival in the standard arm ≤12 months. Currently none of these trials in the non-curative setting reported survival results at 5 years.

When updated results were incorporated in the assessment, clinical benefit of most approved immune checkpoint inhibitors increased, largely due to the statistically significant improvement in the QoL or/and cancer-related symptoms that were not available in the primary reports but reported subsequently. Thus, the score may change when data mature. Our results emphasized the importance of PROs in accurately evaluating the clinical benefit of immune checkpoint inhibitors.

Our study has several limitations. First, toxicities information were extracted from published articles, which often reported only adverse events that occurred in at least 10% of the treated patients, thus, the toxicity grade by ASCO VF may change with complete toxicity information. Second, although we conducted comprehensive research, PROs reports were not available for all approved agents, clinical benefit of these agents may change when PROs report were available. Third, we focused on clinical benefit of immune checkpoint inhibitors, and no comparisons to approved chemotherapy or other agents over a similar time period were conducted.

## Conclusion

In summary, all of the approved immune checkpoint inhibitors based on RCTs meet the ESMO-MCBS threshold for clinical benefit, and two thirds of these approved agents fulfilled the durable benefit thresholds in the updated ASCO VF. This information may be used in future analysis to better define clinical benefits of immunotherapies.

## Data Availability

All relevant data have been provided in the text and on-line supplement. Data sharing: Data extracted from published manuscript is available from the senior author at wozhangsheng@hotmail.com.

## Abbreviations

ESMO-MCBS: European Society for Medical Oncology Magnitude of Clinical Benefit Scale
ASCO-VF: American Society of Clinical Oncology (ASCO) Value Framework
PROs: Patient-reported outcomes
QOL: Quality of life
